# Zyxin is a novel interacting partner for SIRT1

**DOI:** 10.1186/1471-2121-10-6

**Published:** 2009-01-27

**Authors:** Yuki Fujita, Atsushi Yamaguchi, Katsuhiko Hata, Mitsuharu Endo, Naoto Yamaguchi, Toshihide Yamashita

**Affiliations:** 1 Department of Molecular Neuroscience, Graduate School of Medicine, Osaka University, 2-2 Yamadaoka, Suita, Osaka 565-8670, Japan; 2 Department of Neurobiology, Graduate School of Medicine, Chiba University, 1-8-1 Inohana, Chuo-ku, Chiba 260-8670, Japan; 3 Department of Molecular Cell Biology, Graduate School of Pharmaceutical Sciences, Chiba University, 1-8-1 Inohana, Chuo-ku, Chiba 260-8675, Japan

## Abstract

**Background:**

SIRT1 is a mammalian homologue of NAD+-dependent deacetylase sirtuin family. It regulates longevity in several model organisms and is involved with cell survival, differentiation, metabolism among other processes in mammalian cells. SIRT1 modulates functions of various key targets via deacetylation. Recent studies have revealed SIRT1 protects neurons from axonal degeneration or neurodegeneration. Further, SIRT1 null mice exhibit growth retardation and developmental defects, suggesting its critical roles in neurons and development.

**Results:**

To identify novel binding partners for SIRT1 in the central nervous system, we performed yeast two-hybrid screening on human fetal brain cDNA library and found that zyxin is a possible binding partner. SIRT1 and zyxin transcript were both preferentially expressed in developmental mouse brain. Zyxin accumulates in the nucleus where it is co-localized with SIRT1 after treatment with leptomycin B in COS-7 cells. Furthermore, SIRT1 deacetylates zyxin, suggesting SIRT1 could interact with nuclear-accumulated zyxin and modulate its function through deacetylation.

**Conclusion:**

Zyxin could be a novel interacting partner of SIRT1. Zyxin is an adaptor protein at focal adhesion plaque, regulating cytoskeletal dynamics and signal transduction to convey signal from the ECM (extracellular matrix) to the nucleus. Our results raise the possibility that SIRT1 regulates signal transmission from ECM to the nucleus by modulating the functions of zyxin via deacetylation.

## Background

SIRT1 is the mammalian homologue closest to yeast NAD+-dependent deacetylase Sir2 (silent information regulation 2). It was originally identified as a lifespan-extending gene when over-expressed in budding yeast, *Caenorhabditis elegans*, and *Drosophila melanogaster *[1-3]. There are seven homologues of SIRT (named SIRT1-7) in mammals, and SIRT1 is the homologue closest to Sir2. Post-translational modification of proteins, like acetylation, methylation, and phosphorylation, plays a pivotal role in the biological functions [4,5]. Recently, several lines of evidence have indicated that SIRT1 regulates various functions such as cellular survival, differentiation, and metabolism by modulating key targets via deacetylation. These targets include FOXO, Ku70, p53, NF-κB, PGC-1α (PPAR-gamma co-activator 1-alpha) and PPARγ (peroxisome proliferator-activated receptor γ). [6-11]. The knockout of SIRT1 is not embryonic lethal however most null mice die in the perinatal period, exhibiting growth retardation and developmental defects in various tissues including eye, lung, pancreas, heart, and reproductive system[12,13]. This indicates that SIRT1 plays a pivotal role in the development of multiple organ systems. Furthermore, SIRT1 protects neuron from neurodegeneration in cell-based models of Alzheimer's disease, amyotrophic lateral sclerosis (ALS), and Wallerian degeneration [14-16]. However, the molecular mechanism by which SIRT1 functions in the central nervous system (CNS) or in the development remains unclear. In this study, to identify interacting partners for SIRT1, we performed yeast two-hybrid screening on human fetal brain cDNA library. We found that zyxin is one of binding partners for SIRT1.

Zyxin is primarily localized at the focal adhesion plaque complex as an adaptor protein [17,18]. Zyxin contains a proline-rich domain at the N-terminus and three LIM domains at the C-terminus that are cysteine-rich motifs involved in protein-protein interactions. Zyxin interacts with cytoskeleton-related proteins, including α-actinin, Mena/VASP, CRP, and H-warts/LATS1 [19-22], and signaling molecules, including Vav, CasL, and p130^Cas ^[23,24]. Focal adhesion plaques, where cells interact with the extracellular matrix (ECM), form a structure where multiple interactions and signal transduction networks crosstalk. This not only regulates cell adhesion, spreading, and motility but also transduces signals into the nucleus to regulate gene expression, cell proliferation, differentiation, and apoptosis [25,26]. LIM domain proteins, including two related subfamily prototypes zyxin and paxillin, have been implicated in the regulation of cytoskeletal dynamics and signal transduction to convey signals from the ECM to the nucleus at the focal adhesion plaques [17,18]. It was recently reported that zyxin can shuttle between the cytosol and the nucleus, where it could affect transcriptional activity [27]. Furthermore, nuclear-accumulated zyxin executes anti-apoptotic function in cooperation with Akt in myocardiac cells [28,29].

## Results

### Identification of new binding partner for SIRT1

**Figure 1:**
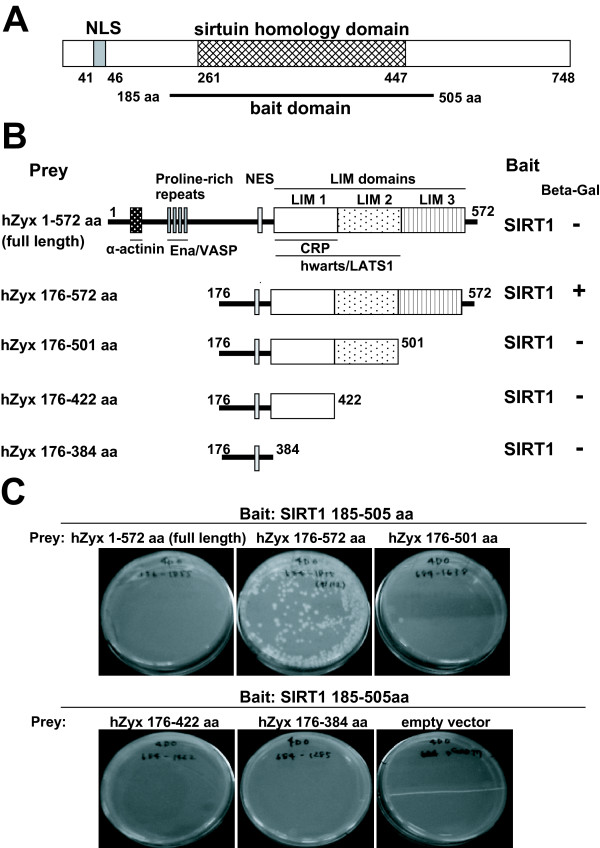
**Interaction between SIRT1 and zyxin in yeast**. (A) Schematic representations of SIRT1 are shown with the sirtuin homology domain. The bar below the SIRT1 structure shows the region used as the bait domain in the yeast two-hybrid system. (B) Schematic diagram of zyxin and its deletion mutants are shown with their domain structures and representative binding partners. (C) In the yeast two-hybrid binding assay, the yeast reporter line (AH109) was co-transformed with plasmids encoding the SIRT1 catalytic domain and various zyxin deletion mutants on a 4-DO (drop out) medium. The line was photographed after 14 days of incubation.

Human SIRT1 encodes 748 amino acids protein with a nuclear localization signal (NLS) at the N-terminus (aa 41–46) and a sirtuin homology domain at the center (aa 261–447); this domain is a conserved catalytic domain for deacetylation (Figure 1A). We performed yeast two-hybrid screening on human fetal brain cDNA library. SIRT1 is reported to perform functions by modulating various targets via deacetylation. We used the human SIRT1 aa 185–505 region, including the conserved sirtuin catalytic domain, as bait. Through this screening, we found several possible interacting partners for SIRT1 (data not shown). Among them, five clones were identical to human zyxin. Recent studies have shown that zyxin can shuttle between the cytoplasm and the nucleus [30] and that it performs anti-apoptotic function in the nucleus [28,29]; these are characteristic features that zyxin shares with SIRT1. Therefore, we decided to perform a functional analysis to study the interaction between SIRT1 and zyxin.

Human zyxin encodes 572 amino acids protein with two major domains: the N-terminal half domain including the proline-rich region and nuclear export signal or nuclear exclusive signal (NES) and three consecutive C-terminal LIM domains (LIM1-3) (Figure 1B). Previous reports have shown the binding regions for α-actinin, Ena/VASP, CRP, and h-warts/LATS1 [19-22], as described in Figure 1B. Since all identified zyxin clones in our screening contained a C-terminal region and since the LIM domain was implicated in protein-protein interaction, we conducted mapping assays concentrating on the LIM domain of zyxin. To map the precise region for zyxin to bind to SIRT1, a yeast two-hybrid assay was performed using the SIRT1 catalytic domain (aa 185–505) as bait and various shorter zyxin fragments as prey. As shown in Figure 1C, colonies appeared only when the yeast reporter line (AH109) containing the SIRT1 catalytic domain was transformed with the plasmid encoding the zyxin aa 176–572 region on a 4-DO (drop out) medium lacking adenine, histidine, leucin, and tryptophan. Furthermore, these colonies exhibit LacZ+ phenotype on β-Gal assay, indicating that the LIM3 domain (aa 501–572) is required for binding to the SIRT1 catalytic domain. Interestingly, we could not detect any colonies where the yeast reporter line (AH109) containing the SIRT1 catalytic domain was transformed with plasmid encoding full-length zyxin on a 4-DO medium. A previous study showed that full-length zyxin interacts with h-warts/LATS1 *in vivo *but not *in vitro*, raising the possibility that LIM1/2 domains are masked in full-length zyxin and some modification might be critical for their interaction [22]. Based on these data, the LIM3 domain is essential for the interaction between SIRT1 and zyxin in yeast; however, we cannot rule out the possibility that some modification might play a pivotal role for the interaction in physiological conditions.

**Figure 2:**
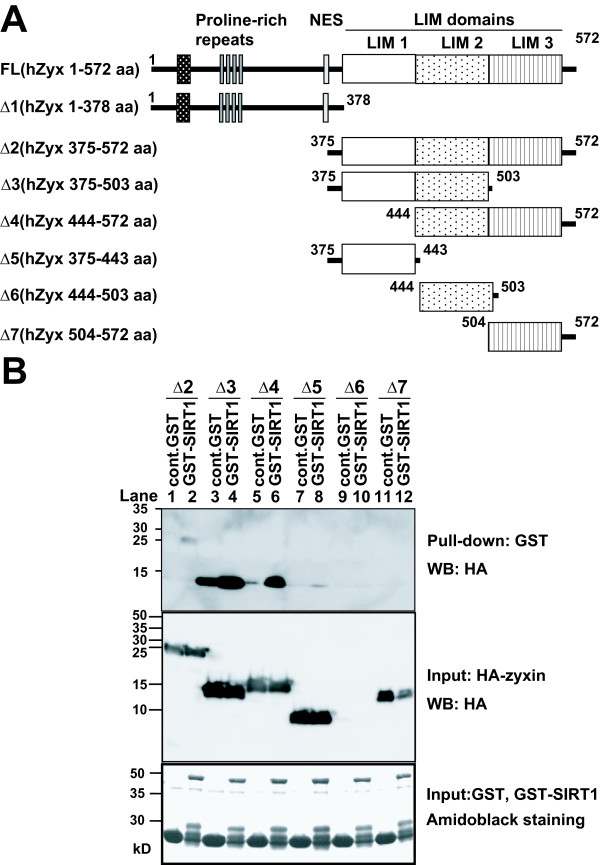
***In vitro *interaction of SIRT1 and zyxin**. (A) Diagram of zyxin full length (FL) and its various deletion mutants. (B) *In vitro *mapping of zyxin domains associates with SIRT1. HEK293T cells expressing the indicated zyxin-deletion mutant were lysed and the resulting lysates were incubated with GST-SIRT1 or control GST proteins immobilized on glutathione sepharose beads. Bound proteins were probed with anti-HA antibody. HA-zyxin or its mutants in lysates were revealed by immunoblotting with anti-HA antibody. The amounts of GST-SIRT1 and control GST proteins were revealed by amido black staining.

To further confirm the region of binding of zyxin to SIRT1, we performed a pull-down assay using bacterially expressed recombinant GST-SIRT1 and various fragments of HA-tagged zyxin expressed in HEK293T cells (Figure 2A). The same amount of GST-SIRT1 fusion proteins bound to glutathione sepharose beads was incubated with various HA-tagged shorter zyxin fragments *in vitro*, and the retained proteins were immunoblotted with anti-HA antibody. As shown in the top panel in Figure 2B, we could detect weak signals in lane 2 and strong signals in lane 6, suggesting that GST-SIRT1 binds to hZyx 375–572 aa (Δ2) weakly and 444–572 aa (Δ4) strongly *in vitro*. We found strong signals in lane 3 in the top panel in Figure 2B, indicating nonspecific binding for hZyx 375–503 aa (Δ3) to GST and/or glutathione-sepharose beads. Therefore, we could not assess the interaction between hZyx 375–503 aa (Δ3) and GST-SIRT1. We also could not assess the interaction between SIRT1 and hZyx 444–503 (Δ6), since we could detect no signals in lanes 9 and 10 in the middle panel of Figure 2B. This suggested that hZyx 444–503 (Δ6) protein might be unstable in HEK293T cells. In addition, we found nonspecific binding of full-length zyxin to GST and/or glutathione-sepharose beads and no signals for the product of hZyx 1–378 aa (Δ1) (Additional file 1), therefore we could not assess the interactions for these proteins. Taken together, these results suggest that zyxin interacts with SIRT1 directly *in vitro *and that the LIM2-3 region of zyxin might be critical for binding to SIRT1 *in vitro*, which is consistent with the result that the LIM3 domain of zyxin is essential for binding to SIRT1 in yeast.

### Sub-cellular localization of SIRT1 and zyxin in COS-7 cells

**Figure 3:**
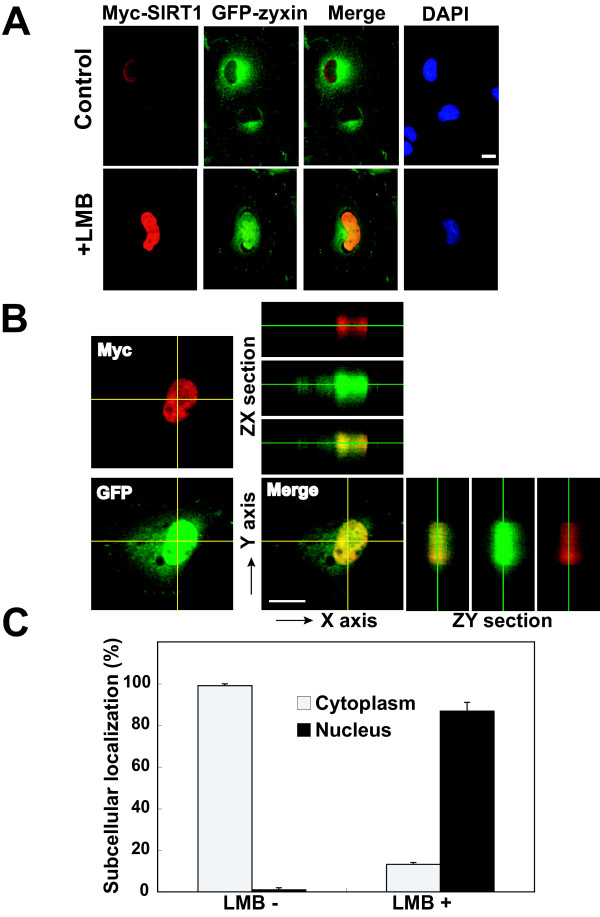
**Leptomycin B treatment causes nuclear accumulation of zyxin and results in co-localization of SIRT1 with zyxin**. (A) COS-7 cells transfected with plasmids encoding HA-SIRT1 and GFP-zyxin were cultured for 24 h, in the presence or absence of 20 nM Leptomycin B during the last 6 h. Cells were stained with anti-Myc. Bars, 20 μm. (B) Confocal images show colocalization of SIRT1 and zyxin in nucleus in the presense of LMB. Bar, 10 μm. (C) Zyxin localization was classified in COS-7 cells expressing HA-SIRT1 and GFP-zyxin. "Cytoplasm" indicates COS-7 cell exhibiting GFP-zyxin only in the cytosol. "Nucleus" indicates COS-7 cell exhibiting nuclear-accumulated GFP-zyxin. Results (%) are the mean ± SD from three independent experiments.

Next, we examined the sub-cellular localization of SIRT1 and zyxin in mammalian cells by an immunocytochemistry assay. COS-7 cells were co-transfected with plasmids expressing Myc-tagged SIRT1 and GFP-zyxin. As shown in Figure 3A, SIRT1 is primarily localized in the nucleus with a diffused pattern. GFP-zyxin, diffusedly localized in the cytosol with some puncta, accumulates in the nucleus after treatment with leptomycin B (LMB), an inhibitor of CRM1-mediated nuclear exportation (Figure 3A). The treatment with LMB induced the accumulation of GFP-zyxin in the nucleus, where it is co-localized with SIRT1 (Figure 3A). To further confirm the co-localization between SIRT1 and GFP-zyxin, we examined the co-localization using ZX and ZY sections taken by scanning confocal microscopy. As in Figure 3B, Myc-tagged SIRT1 and GFP-zyxin are clearly co-localized in the nucleus. We then quantified the LMB-induced nuclear accumulation of zyxin. LMB treatment remarkably increases the number of COS-7 cells exhibiting nuclear-accumulated GFP-zyxin (Figure 3C). Taken together, these results suggest that zyxin, basically localized in the cytosol, translocates into the nucleus, where it is co-localized with SIRT1 in COS-7 cells.

### Interaction between SIRT1 and zyxin in mammalian cells

**Figure 4:**
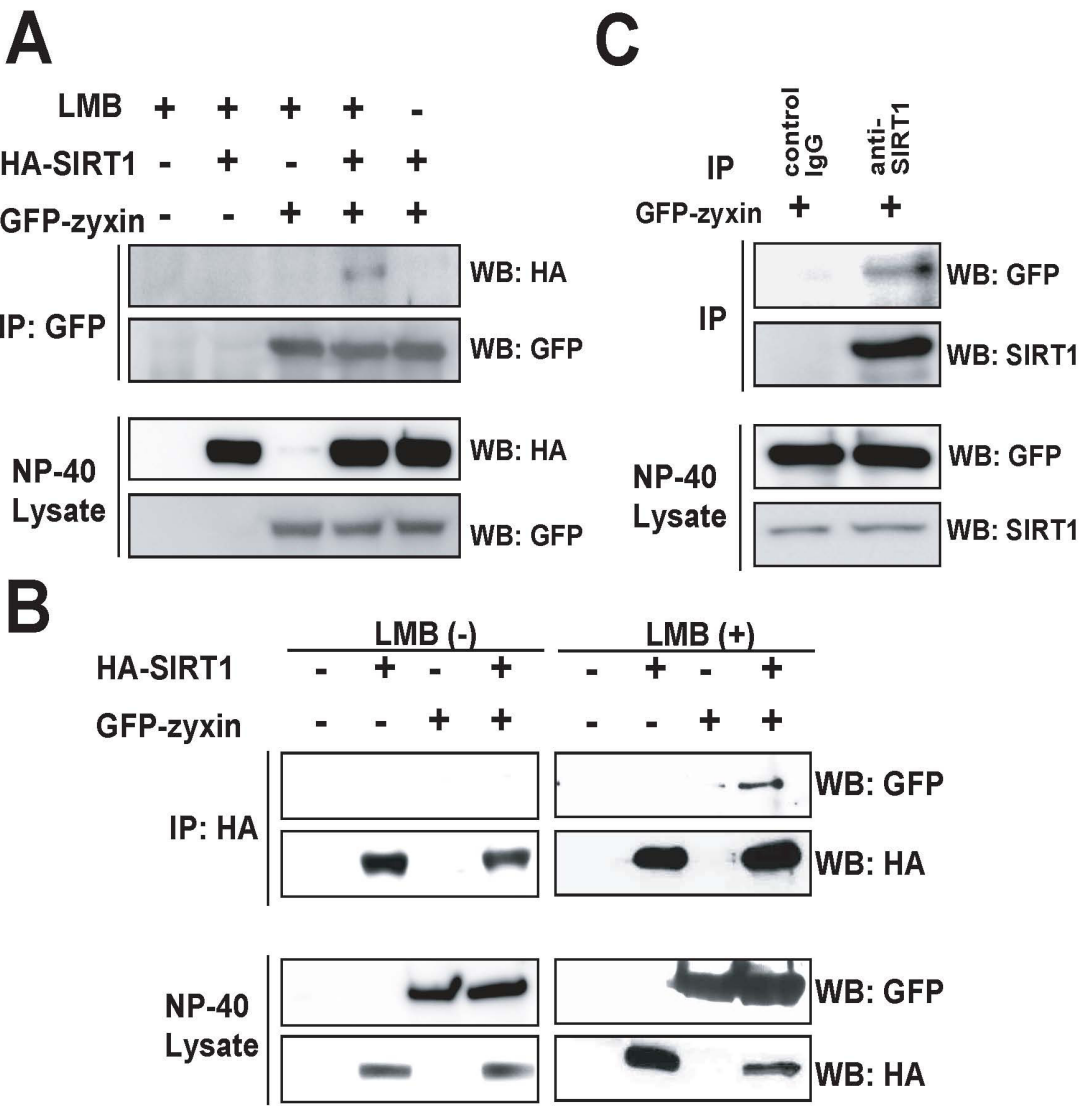
**Interaction of SIRT1 with zyxin in mammalian cells**. (A, B) Co-immunoprecipitation of SIRT1 and zyxin HEK293T cells transiently transfected with the indicated plasmids and treated with 20 nM of leptomycin B (LMB) for 6 h. Cell lysates were immunoprecipitated with the anti-GFP antibody (A) or anti-HA antibody (B). The immunoprecipitates were immunoblotted with a reciprocal antibody. (C) Endogenous SIRT1 interacts with GFP-zyxin. The lysates of HEK293T cells expressing GFP-zyxin were immunoprecipitated with anti-SIRT1 antibody or mouse IgG. The immunoprecipitates were immunoblotted with anti-GFP antibody.

To confirm the interaction between SIRT1 and zyxin in mammalian cells, we performed a co-immunoprecipitation assay using HEK293T cells. HEK293T cells were transiently co-transfected with expression vectors for HA-tagged SIRT1 and GFP-zyxin individually or in combination, in the presence or absence of LMB. Cell lysates were immunoprecipitated with anti-GFP or anti-HA antibody, followed by Western blot analysis with a reciprocal antibody. HA-tagged SIRT1 was detected in the immunoprecipitates with anti-GFP antibody only in the presence of LMB, but not in the absence of LMB (Figure 4A). Furthermore, GFP-zyxin was also detected in the immunoprecipitates with anti-HA antibody only in the presence of LMB, but not in the absence of LMB (Figure 4B). These data suggest that LMB induces the accumulation of GFP-zyxin in the nucleus, leading to the interaction between HA-tagged SIRT1 and GFP-zyxin probably in the nucleus. This is consistent with our morphological data that indicate SIRT1 and zyxin are co-localized in the nucleus with LMB treatment.

Next, to confirm the interaction between endogenous SIRT1 and GFP-zyxin, we performed a co-immunoprecipitation assay using HEK292T cells expressing SIRT1 endogenously. HEK293T cells were transfected with GFP-zyxin expressing plasmid, and cell lysates were immunoprecipitated with anti-SIRT1 antibody or control IgG, followed by Western blot analysis with anti-GFP antibody (Figure 4C). GFP-zyxin was detected in the immunoprecipitates with anti-SIRT1 antibody, but not in those with control IgG, indicating the interaction between endogenous SIRT1 and exogenously overexpressed GFP-zyxin. Taken together, these results suggest that SIRT1 and zyxin, both shuttling between cytosol and nucleus, could interact primarily in the nucleus in HEK293T cells.

### Expression profiles of SIRT1 and zyxin

**Figure 5:**
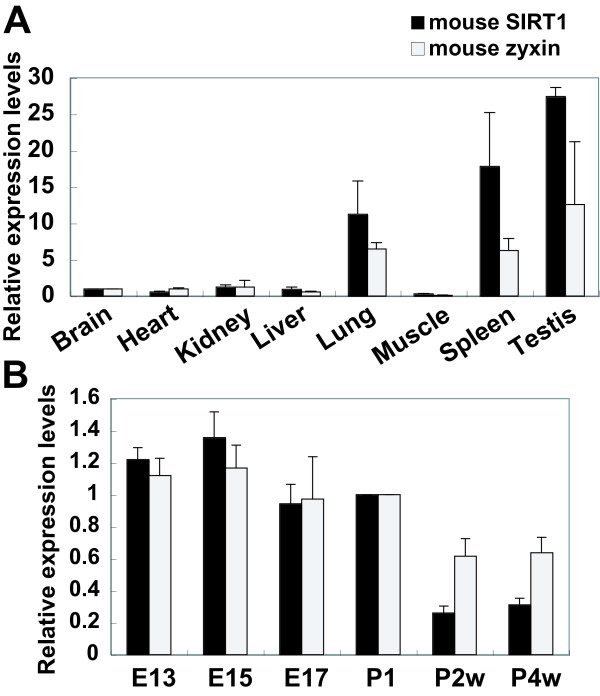
**Relative expression levels of SIRT1 and zyxin mRNA in various mouse tissues and developmental stages**. (A) Quantitative real-time PCR expression profiles of mouse cDNA from different tissues. The expression levels of SIRT1 and zyxin relative to those of GAPDH were assessed by ΔΔCt method. Results are the mean ± S.E from three independent reactions. (B) Real-time PCR expression profiles of mouse cDNA from whole brain at different developmental stages. The expression levels of SIRT1 and zyxin mRNA are represented as relative fold as compared with the value in brain(A) and P1(B). E13, 15, 17; Embryonic day 13, 15, 17. P1, P2w, P4w: Post natal day 1, 2 weeks, 4 weeks.

To examine the expression profiles of SIRT1 and zyxin transcript, we performed real-time PCR assay using total RNAs derived from various adult mouse tissues. As shown in Figure 5A, the expression pattern of zyxin transcript is similar to that of SIRT1, with preferential expression in the lung, spleen, and testis.

Then, to investigate the expression profile of SIRT1 and zyxin transcript in the developmental brain, we performed real-time PCR assay using total RNAs derived from mouse brains at various developmental stages. The expression pattern of zyxin transcript in the developmental brain is also similar to that of SIRT1, with preferential expression at the E13-P1 stage (Figure 5B).

### SIRT1 deacetylates zyxin *in vitro* and *in vivo*

**Figure 6:**
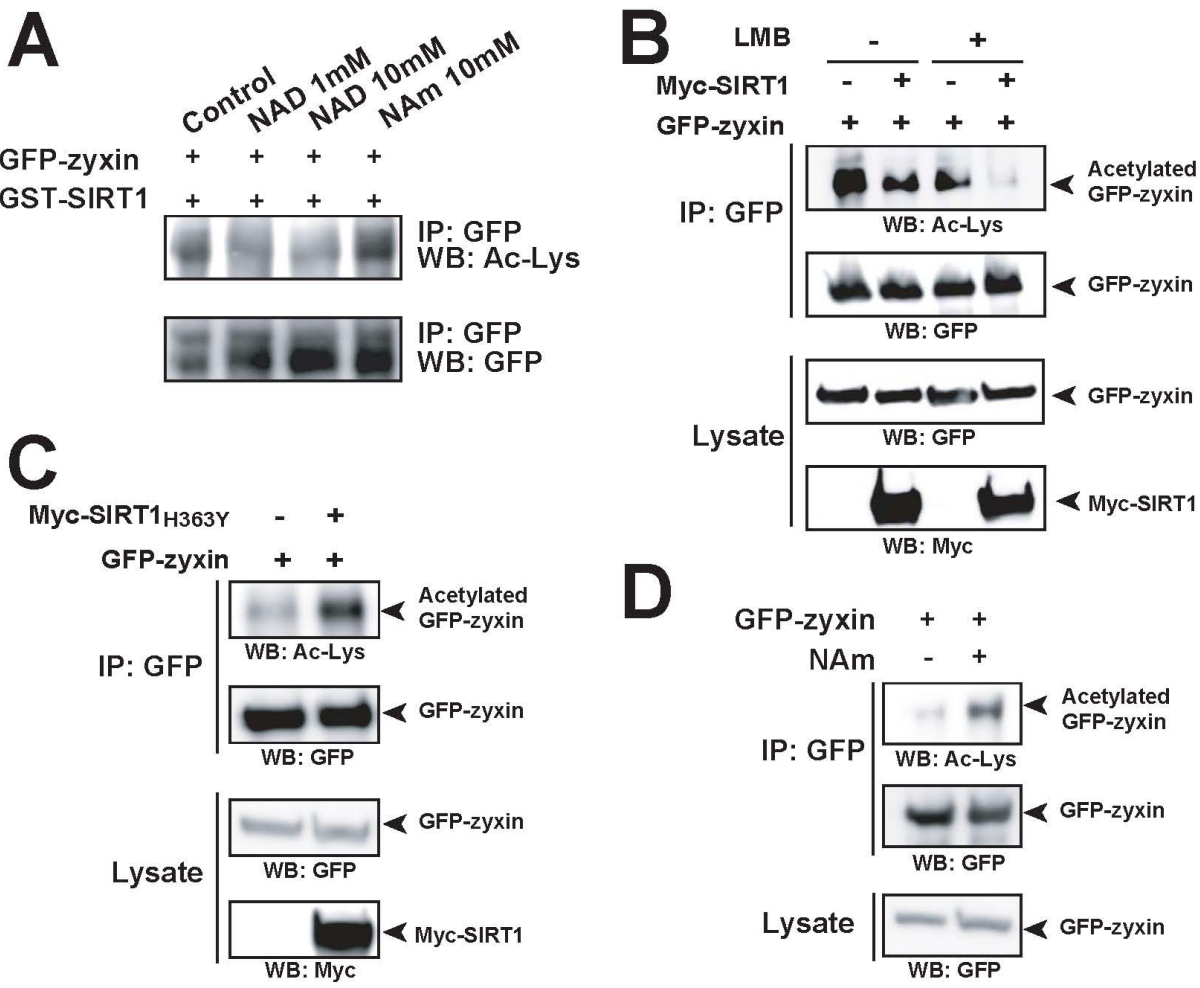
**SIRT1 deacetylates zyxin *in vitro *and *in vivo***. (A) SIRT1 deacetylates zyxin *in vitro*. GFP-zyxin, immunoprecipitated using anti-GFP antibody, was added with recombinant GST-SIRT1 in the presence or absence of NAD or nicotinamide (NAm). The acetylation levels of zyxin were determined using anti-acetylated lysine antibody. (B) SIRT1 deacetylates zyxin in mammalian cells. COS-7 cells were transfected with plasmids expressing GFP-zyxin and Myc-SIRT1 and incubated for 6 h in the presence or absence of leptomycin B. GFP-Zyxin was immunoprecipitated with anti-GFP antibody, and the acetylation levels of zyxin were determined using anti-acetylated lysine antibody. (C) SIRT1 H363Y does not affect the acetylation levels of zyxin. COS-7 cells were transfected with plasmids expressing GFP-zyxin and Myc-SIRT1 H363Y. GFP-Zyxin was immunoprecipitated with anti-GFP antibody, and the acetylation levels of zyxin were determined using anti-acetylated lysine antibody. (D) HEK 293T cells expressing GFP-zyxin were treated for 24 h in the presence or absence of NAm. GFP-zyxin was immunoprecipitated using GFP antibody, and the acetylation levels of zyxin were determined using anti-acetylated lysine antibody.

SIRT1 is an NAD+-dependent protein deacetylase that targets a wide variety of proteins to modulate their functions through deacetylation. Since we used the catalytic domain of SIRT1 as bait in our screening, we sought to investigate whether SIRT1 deacetylates zyxin. We first performed *in vitro *deacetylation assay using bacterially expressed recombinant GST-SIRT1 and GFP-zyxin expressed in HEK293T cells. Immunoprecipitated GFP-zyxin with anti-GFP antibody was incubated in the reaction buffer containing bacterially expressed GST-SIRT1, in the presence of NAD+ or SIRT1 inhibitor, nicotinamide (NAm). The samples were resolved on a SDS-PAGE, and the acetylation status was monitored by immunoblotting with anti-Ac-Lys antibody, a specific antibody for acetylated lysine. As shown in Figure 6A, the signals for acetylated GFP-zyxin decreased in an NAD+-dependent manner; this was abolished in the presence of NAm. These results indicate that SIRT1 can deacetylate zyxin directly in an NAD+-dependent manner *in vitro*.

We next examined whether SIRT1 mediates the deacetylation of zyxin *in vivo*. COS-7 cells were co-transfected with expressing plasmids encoding Myc-tagged SIRT1 and GFP-zyxin in the presence or absence of LMB, and cell lysates were immunoprecipitated with anti-GFP antibody followed by Western blot analysis using anti-Ac-Lys antibody to monitor acetylation levels. As shown in Figure 6B, the signals for acetylated GFP-zyxin were remarkably reduced in the presence of Myc-tagged SIRT (first panel, lanes 2 and 4) as compared with the control (first panel, lanes 1 and 3), suggesting that SIRT1 can deacetylate zyxin *in vivo*. The signals for acetylated GFP-zyxin without LMB treatment are stronger as compared to those with LMB treatment (Figure 6B, first panel), while the amounts of total GFP-zyxin protein in immunoprecipitates are comparable (Figure 6B, second panel). This suggests that SIRT1 could deacetylate nuclear-accumulated zyxin. To confirm the specificity of deacetylation by SIRT1, we performed an *in vivo *deacetylation assay using SIRT1 H363Y, a loss-of-function mutant [6,9]. The signals for acetylated GFP-zyxin were remarkably enhanced by SIRT1 H363Y overexpression (Figure 6C), indicating the specificity for deacetylation by SIRT1. To strengthen this result, we then examined the effect of SIRT1 inhibitor, nicotinamide (NAm), in an *in vivo *deacetylation assay. HEK293T cells were transfected with plasmids expressing GFP-zyxin, with or without NAm treatment for 24 h, and GFP-zyxin was immunoprecipitated with anti-GFP antibody. The deacetylation levels were monitored by a Western blot analysis with anti-Ac-Lys antibody. The signals for acetylated GFP-zyxin were remarkably enhanced in the presence of NAm (Figure 6D), indicating the specificity of deacetylation by SIRT1. Taken together, these results clearly show that in mammalian cells, SIRT1 can modulate the deacetylation levels of zyxin, most likely in the nucleus.

## Discussion

The present study shows that zyxin is a novel interacting partner for SIRT1. SIRT1 and zyxin are co-localized in the nucleus after LMB treatment, suggesting that nuclear-accumulated zyxin interacts with SIRT1. SIRT1 and zyxin transcript are both preferentially expressed in developing mouse brain and various adult tissues, including lungs, spleen, and testis. Furthermore, SIRT1 deacetylates zyxin, especially after LMB treatment, raising the possibility that SIRT1 could modulate zyxin's functions in the nucleus via deacetylation. Zyxin-null mice exhibit almost no abnormal phenotype, probably due to genetic redundancy [31]. On the other hand, SIRT1-null mice show growth retardation and developmental defects in various tissues [12,13], which suggests a critical role of SIRT1 in the developmental stage. Based on these reports, our data could raise the possibility that zyxin is one of the downstream effectors necessary for SIRT1 to execute some biological functions in the developing brain and various adult tissues.

There is an apparent contradiction between the yeast and mammalian results. Apparently SIRT1 does not bind to full length zyxin in yeast because of some hypothesized masking of the LIM3. However, the mammalian cell experiments do show apparent association between full-length zyxin and SIRT1. As mentioned above, a previous study showed that full-length zyxin interacts with h-warts/LATS1 *in vivo *but not *in vitro*, raising the possibility that LIM1/2 domains are masked in full-length zyxin and some modification might be critical for their interaction [22]. Therefore we also hypothesize some modification would be required for full-length zyxin to bind to SIRT1 in mammalian cells.

Then what is the biological function of the interaction between SIRT1 and zyxin? Cell survival effect is one of the features shared by these proteins. SIRT1 can protect neurons from oxidative stress in mammalian cells [32], neurotoxicity in cell-based models for AD/tauopathies, ALS [16], and Wallerian degeneration [14]. SIRT1 expression levels are induced in mouse models of neurodegeneration [16], and moderate heart-specific overexpression of SIRT1 in mice delays aging of the heart by conferring resistance to oxidative stress [33]. On the other hand, nuclear-accumulated zyxin can protect cardiomyctes from oxidative stress [28]. These reports raise the possibility that the interaction of these proteins could be implicated in cellular survival, especially in the brain and heart, during physiological senescence. Since zyxin could affect transcriptional activity [27] and since nuclear-accumulated zyxin executes anti-apoptotic function cooperating with Akt in the nucleus [28], it is possible SIRT1 could modulate these functions of zyxin via deacetylation. Considering that SIRT1-null mice exhibit developmental defects with frequent exencephaly and retinal phenotype with abnormal proliferation [12,13], it is possible that the interaction of these proteins could be implicated in cellular survival pathways in the developmental stage.

In addition, shuttling between the cytosol and the nucleus is a characteristic feature shared by SIRT1 and zyxin. In this study, it was not determined whether the acetylation status of zyxin affects its cellular localization or whether deacetylated zyxin is retained in the nucleus. Recently, it was reported that nuclear zyxin, phosphorylated by Akt, interacts with acinus-S to prevent apoptosis and 14-3-3γ plays a pivotal role in the nuclear translocation of zyxin [29]. The morphological studies in the present study show that zyxin is localized primarily in the cytosol with puncta and it accumulates in the nucleus only with the LMB treatment, but not with insulin-like growth factor (IGF-1) or LY294002 treatment, an inhibitor of PI 3-kinase, in COS-7 cells (data not shown). This suggests that some unknown stimuli other than the IGF-1-Akt-related pathway could play a critical role for zyxin to translocate into the nucleus. The nuclear accumulation of zyxin is not induced by co-expression with SIRT1, suggesting that it is unlikely SIRT1 enhances the accumulation and/or translocation of zyxin into the nucleus. Since the sub-cellular localization of SIRT1 differs based on cell type and differentiation [12,32-34], it is possible that SIRT1 could interact with zyxin in the cytosol depending on the cell type and differentiation.

ECM, beyond scaffolding functions, is responsible for transmitting environmental signals into cells, thereby essentially affecting all aspects of cell life, including its proliferation, differentiation, and death [35]. Since zyxin is considered to convey signals from ECM into the nucleus at the focal adhesion plaques, we now assume that SIRT1 could regulate the signal transmission from ECM into the nucleus by modulating zyxin's functions via deacetylation, thereby reflecting its biological functions.

Finally as described in above, several functional significances could be assumed for the interaction, including the cell survival effect especially in development and/or regulation of signal transmission from ECM into the nucleus. Actually we had performed experiments to investigate whether the interaction of these proteins could affect the apoptosis under stress condition or the transcriptional activity of zyxin in luciferase reporter gene assay (data not shown). Thus far, we could not obtain the result to indicate the biological significance of the interaction in such simple systems. Additional experiments are required to clarify the functional significance of the interaction in the future.

## Conclusion

In conclusion, zyxin could be a novel interacting partner of SIRT1. Zyxin is an adaptor protein at focal adhesion plaque, regulating cytoskeletal dynamics and signal transduction to convey signal from the ECM (extracellular matrix) to the nucleus. Our results raise the possibility that SIRT1 regulates signal transmission from ECM to the nucleus by modulating the functions of zyxin via deacetylation.

## Methods

### Cell culture and transfection

Human embryonic kidney cell line HEK 293T cells and African green monkey kidney fibroblast like cell line COS-7 cells were cultured in DMEM (Gibco) supplemented with 10% fetal bovine serum (FBS) and antibiotics (50 U penicillin-streptomycin) at 37°C in a 5% CO_2 _atmosphere inside a humidified incubator. Transient transfection was performed using Lipofectamine 2000 (Invitrogen) according to the manufacturer's instructions.

### Plasmid constructs

The FLAG-tagged mammalian expression vectors encoding wild-type (wt) human SIRT1 or a mutant catalytically inactive SIRT1 [6], SIRT1 H363Y, were obtained from Addgene (USA). The mammalian expression vectors encoding the Myc-tagged form of human SIRT1 were generated by sub-cloning from SIRT1 wt or SIRT1 H363Y cDNA fragments into the Sac1-Sal1 site of the pMyc-C1 mammalian expression vector containing the Myc epitope tag [36]. SIRT1 wt cDNA were sub-cloned into pGEX-6P-3 (GE Healthcare) for bacterial GST fusion expression.

pEGFP-C1-zyxin was provided by Dr. Saya (Kumamoto University School of Medicine, Japan). For the pull-down assay, cDNA fragments encoding full-length and deletion mutants of zyxin (as illustrated in Figure 2A) were amplified by PCR from pEGFP-zyxin and sub-cloned into pHA-C1 mammalian expression vector containing the HA epitope tag [36]. All constructs were verified by DNA sequencing.

### Antibodies

Monoclonal anti-HA (HA-7; Sigma-Aldrich), anti-c-Myc (9E10; Santa Cruz Biotechnology, Inc.), and anti-SIRT1 (2G1/F7; Upstate), polyclonal anti-Acetylated lysine (Cell Signaling technology) were used.

### Yeast two-hybrid screening

Yeast two-hybrid screening was conducted using Matchmaker GAL4 two-hybrid system 3 (Clontech). The region containing the deacetylation domain of SIRT1 (amino acid (aa) 185–505) was generated by PCR, subcloned downstream of the GAL4 DNA-binding domain in pGBKT7, and introduced into the yeast strain AH109 as bait. The human fetal brain cDNA library in pGADT7 was introduced into the yeast strain Y187. These two yeast strains were combined according to the yeast mating protocol recommended by the manufacturer. Diploid cells were screened for growth on SD agar plates lacking adenine, histidine, leucine, and tryptophan (4 drop out).

To confirm the yeast two-hybrid assay results, the yeast strain AH109 was retransformed with pGBKT-SIRT1/bait (aa 185–505 of SIRT1) and histidine and adenine positive clones. Then, the positive clones were further evaluated for beta-GAL expression by colony-lift filter assay.

The plasmids were isolated from a colony showing beta-GAL activity using an alkaline solution (3% SDS, 0.2 M NaOH), transformed into bacteria by heatshock or electroporation, and then DNA sequenced. The clones encoding the partial protein fragments of zyxin were determined.

### Pull-down assay

Transiently transfected HEK 293T cells were lysed with a lysis buffer (0.5% NP-40, 20 mM HEPES, 150 mM NaCl, 1 mM EDTA, protease inhibitor cocktail (Roche)). Cell lysates were pre-cleared with GST immobilized on glutathione-sepharose 4B (GE Healthcare) for 1 h. GST fusion protein (10 μg) was immobilized on glutathione-sepharose beads at 4°C for 4 h. Then the glutathione-sepharose beads were incubated with cell lysate in 500 μL of the lysis buffer for 4 h at 4°C. After washing with a wash buffer (0.1% NP-40, 20 mM HEPES, 150 mM NaCl, 1 mM EDTA), the bound proteins were eluted by boiling for 5 min in a sample buffer (50 mM Tris-HCl (pH 6.8), 2% SDS, 8% glycerol, 4% mercaptoethanol, 0.04% bromophenol blue), and analyzed by western blot analysis.

### RNA extraction, reverse transcription, and real-time PCR

Isolated mouse brains at various ages and various normal tissues were flash-frozen in liquid nitrogen and stored at -80°C until RNA purification. Total RNA was extracted using Trizol (Invitrogen) according to the manufacturer's instructions. RNA (2 μg) was reverse transcribed to produce cDNA using Ready-To-Go RT-PCR beads (GE Healthcare).

Real-time PCR was performed using SYBR GreeenER qPCR SuperMis for ABI PRISM (Invitrogen). All real-time PCR assays were performed in triplicate using a 7300 Real-Time PCR System (Applied Biosystems) as follows: 50°C for 2 min, 95°C for 10 min, 40 cycles at 95°C for 20 s and at 60°C for 1 min. Primers for the mouse Sir2α (SIRT1) mRNA were designed as described previously [34]. Primers for mouse zyxin were designed using Primer Express version 3.0 (Applied Biosystems). For standardization of relative mRNA expression, rodent GAPDH primers were used. The specificity of each primer set was determined with a pre-test showing the specific amplification for a specific gene by gel visualization and sequencing. The results of cycle threshold values (Ct values) were calculated by the ΔΔCt method to obtain the fold differences.

### Immunofluorescence

COS-7 cells transfected with plasmids encoding GFP-zyxin and/or HA-SIRT1 were cultured for 42 h and treated with or without Leptomycin B (LMB) for the next 6 h. Then, the cells were fixed with 4% paraformaldehyde for 1 h and then rehydrated by PBS. Cells were permeabilized and non-specific sites were blocked by incubating with PBS containing 0.5% Triton X-100 and 5% bovine serum albumin (BSA). The cells were incubated with antibodies diluted in a blocking solution for 1 h at 4°C. Then, the cells were washed in PBS and incubated with secondary antibodies, Alexa fluor, for 1 h at room temperature. After immuno staining, the slides were mounted with a fluorescent mounting medium (DakoCytomation). Confocal images were obtained using a Fluoview FV1000 (Olympus) laser scanning microscope. XZ and YZ sections were created when all Z-series sections at 0.50 μm intervals were merged.

### Immunoprecipitation

HEK293T cells were transfected with plasmids encoding GFP-zyxin and/or HA-SIRT1 using Lipofectamine 2000 reagent as previously described. The transfected cells were cultured for 42 h and treated with or without leptomycin B (LMB) for the next 6 h. To immunoprecipitate the expressed GFP-tagged proteins, the cells were lysed in a lysis buffer (0.5% NP-40, 20 mM HEPES (pH 8.0), 150 mM NaCl, 1 mM EDTA, protease inhibitor cocktail (Roche)). The whole-cell lysates were pre-cleared with rProtein A sepharose Fast Flow (GE Healthcare) at 4°C for 1 h. The supernatants were incubated at 4°C for 4 h with anti-GFP antibody or anti-HA antibody and rProtein A sepharose. After washing three times in a wash buffer, the immunoprecipitates were boiled in a sample buffer containing 12% beta-mercaptoethanol for 5 min and subjected to western blot analysis.

### Western blot analysis

Western blot analysis was modified as described previously [37]. In brief, cell lysates or immunoprecipitated proteins were separated on SDS-PAGE and transferred onto polyvinylidene difluoride membranes (Millipore). The membrane was blocked with 5% nonfat dry milk in PBS containing 0.05% Tween 20 (PBS-T) and incubated for 2 h at room temperature with the first antibody diluted in PBS-T containing 1% nonfat dry milk. After washing with PBS-T, the membrane was incubated with horseradish peroxidase-linked anti-mouse IgG antibody or anti-rabbit IgG antibody (Cell Signaling Technology). For detection, an ECL chemiluminescence system (GE Healthcare) was used.

### Deacetylation assay

For the *in vitro *deacetylation assay, zyxin was immunoprecipitated and incubated with GST-SIRT1. The deacetylation reaction was performed as reported previously [38]. COS-7 or HEK293T cells were transfected with plasmids encoding GFP-zyxin and/or Myc-SIRT1. The cells were cultured for 48 h in the presence or absence of LMB during the last 6 h. The cells were lysed with an RIPA buffer (20 mM Tris-HCl (pH 7.4), 150 mM NaCl, 1 mM EDTA, 0.1% SDS, 1% Triton X-100, 1% sodium deoxycholate, protease inhibitor cocktail (Roche)). Lysates were immunoprecipitated with anti-GFP antibody and the acetylation levels of zyxin were detected by anti-acetylated lysine antibody.

## Authors' contributions

YF and AY carried out the molecular genetic studies, participated in the design of the study and drafted the manuscript. KH and ME participated in the design of the study and performed the morphological analysis. NY and TY conceived of the study, and participated in its design and coordination. All authors read and approved the final manuscript.

## Supplementary Material

Additional file 1 **Full-length zyxin and deletion mutant zyxin (hZyx 1–378 aa (Δ1)).** The data show nonspecific binding of full-length zyxin to GST and/or glutathione-sepharose beads and no signals for the product of hZyx 1–378 aa (Δ1). HEK293T cells expressing the full-length or indicated deletion mutant of zyxin were lysed and the resulting lysates were incubated with GST-SIRT1 or control GST proteins immobilized on glutathione sepharose beads. Bound proteins were probed with anti-HA antibody (upper left panel). HA-zyxin or its mutants in lysates were revealed by immunoblotting with anti-HA antibody (upper right panel). The amounts of GST-SIRT1 and control GST proteins were revealed by amido black staining (lower panel). Click here for file
